# Optimizing Edible Sorghum Bowls: Effects of Roasting and Edible Flower Powder Enhancement on Technological, Nutritional, Antioxidant, and Functional Properties

**DOI:** 10.1155/ijfo/1771084

**Published:** 2025-01-06

**Authors:** Devatha Manivel, Raajeswari Paramasivam, Swarup Roy

**Affiliations:** ^1^Department of Food Science and Nutrition, Avinashilingam Institute for Home Science and Higher Education for Women, Coimbatore, Tamil Nadu, India; ^2^Department of Food Technology and Nutrition, School of Agriculture, Lovely Professional University, Phagwara, Punjab, India

**Keywords:** biodegradable, functional enhancement, hibiscus, less cytotoxicity, property analysis, rose, sorghum bowl

## Abstract

The widespread reliance on single-use plastics (SUPs) has fostered a global throwaway culture, especially in the food packaging industry, where convenience and low cost have driven their adoption, posing serious environmental threats, particularly to marine ecosystems and biodiversity. Edible and ecofriendly packaging made from millet, specifically sorghum (*Sorghum bicolor* (*L.*) Moench), is a promising solution to mitigate SUP consumption and promote sustainability. This study explores the development of edible sorghum bowls, enhanced through roasting and incorporating 3 g of hibiscus and rose flower powders. The standardized sorghum bowl was analyzed for nutritional value; optical, technological, functional, and mechanical properties; and shelf life, and the results were discussed. The bowls, 18.5 g of average weight, dimensions of 10.2 cm, and a thickness of 3 mm, were baked in a unique bowl-shaped mold at 80°C for 7 min. Enhancing the bowls with flower powder improved their optical properties and nutrient content. The addition of flower powder also increased phytochemical levels, according to qualitative analysis, while roasting sorghum reduced tannin and phytic acid content. The IC50 values revealed that hibiscus (47.74 mg/mL) and rose (39.87 mg/mL) enrichment boosted antioxidant activity. Sensory evaluations favored roasted bowls across all attributes, while Fourier transform infrared spectroscopy (FTIR) and thermogravimetric analyzer (TGA) analyses confirmed significant structural changes. The enhanced bowls exhibited greater hardness and hold hot or cold snacks for 90 min without compromising structural integrity. Additionally, these bowls demonstrated an extended shelf life, low microbial count (1 × 10^1^CFU/g), reduced toxicity (3%–10% mortality in brine shrimp assays), and complete biodegradation within 15 days in wet soil. These findings indicate that sorghum-based edible bowls present a nutritious, viable, less toxic alternative to SUPs, appealing to a broad demographic, especially in the food and tourism sector, and contributing to environmental conservation by reducing plastic waste and suitable for wide consumption.

## 1. Introduction

The pervasive use of single-use plastics (SUPs) has fostered a global throwaway culture, especially in the food packaging industry, where convenience, low cost, and durability have driven their widespread adoption. This trend has resulted in a significant escalation of plastic waste generation, posing severe environmental threats, particularly to marine ecosystems and biodiversity [1, 2]. The United Nations Environment Programme (UNEP) estimates that approximately 300 million tons of plastic waste are generated annually, with only 9% being recycled, 12% incinerated, and a staggering 79% accumulating in landfills or the natural environment [3]. Since 1950, global plastic production has skyrocketed from 1.5 million tons to around 370 million tons by 2019. Asia leads with 51% of production, followed by North American Free Trade Agreement (NAFTA) countries (19%) and Europe (16%). If current trends continue, plastic litter in landfills and natural ecosystems could reach 12 billion tons by 2050 [4, 5]. The persistence of plastic waste due to its nonbiodegradable nature, including items like disposable bowls, exacerbates these challenges and underscores the urgent need for sustainable alternatives that can replace conventional plastic products and reduce the environmental footprint of SUPs. Recent developments in edible packaging have shown promising potential to not only replace conventional plastic materials but also to offer added nutritional and functional advantages. Research has explored a variety of plant-based materials, such as seaweed, rice bran, and potato starch, for the formulation of biodegradable edible packaging and coatings that effectively enhance food shelf life by providing protective barriers against spoilage and microbial contamination [6]. Biodegradable films provide a basis for ecofriendly alternatives, highlighting the importance of creating substitutes that are low-density, cost-effective, and easy to process, while also supporting advanced packaging designs [7]. The global market for biodegradable packaging is projected to grow at a compound annual growth rate (CAGR) of 15.3% from 2021 to 2028, driven by rising environmental concerns and stringent regulations on SUPs. Sixty-four percent of consumers are willing to pay a premium for food products that are both sustainable and beneficial to health [8, 9]. Edible cutlery provides an innovative solution to plastic pollution by catering to the growing consumer demand for ecofriendly products. A notable development in this area is the creation of edible packaging, such as millet-based bowls, which serve as biodegradable alternatives to SUPs and contribute to reducing environmental impact [10, 11]. Millets, small-seeded cereals from the Poaceae family, are traditionally grown in arid and semiarid regions and have been staple foods in low-income communities [12]. Despite their rich nutritional content, including high dietary fiber, their potential in creating functional foods remains largely underexploited. Standardizing millet-based commercial products is crucial for unlocking their full potential in the global food industry [13].

Sorghum (*Sorghum bicolor* (L.) Moench), a prominent member of the millet family, is the fifth most cultivated cereal globally, valued for its resilience in warm, dry climates [14]. Sorghum, available in different varieties such as white, yellow, and red, is rich in bioactive compounds that offer a range of health benefits, including immune modulation, antioxidant activity, and anti-inflammatory effects [15]. The global market for roasted sorghum products is expanding, driven by their gluten-free nature and appealing flavor profile [16]. Recent research underscores the potential of sorghum and other millets in developing value-added products for health-conscious consumers, yet this potential remains largely untapped [17, 18]. Developing functional foods and biodegradable materials from millets and sorghum presents a significant research gap with both economic and environmental benefits [19]. The growing consumer interest in bioactive compounds has also boosted the popularity of edible flowers, known not only for their ornamental value but also for their nutritional and medicinal properties [20–22]. Rose (*Rosa damascena* Mill.) and hibiscus (*Hibiscus rosa-sinensis* L.) are particularly noteworthy. Rose is valued in traditional and modern medicine for its essential oils, which have antioxidant, antibacterial, antimicrobial, and anti-inflammatory properties and are used to treat conditions like menstrual bleeding, inflammatory bowel disease (IBD), and gastroesophageal reflux [23, 24]. Hibiscus, recognized for its therapeutic benefits, adds a natural sour flavor and vibrant color to foods and beverages and exhibits significant antibacterial, antioxidant, lipid-modulating, and insulin resistance-reducing effects [25, 26]. Rose and hibiscus are rich sources of bioactive phytochemicals, contributing significantly to their health-promoting properties. Rose contains a variety of flavonoids (such as quercetin, kaempferol, and apigenin), flavan-3-ols (catechin), anthocyanins, proanthocyanidins (procyanidin), and phenolic acids (gallic and ellagic acid), along with stilbenes like resveratrol. Similarly, hibiscus provides phenolic acids, flavonoids, organic acids, lutein, tannins, and various anthocyanins, further enhancing its functional potential [23–26]. Combining roasted sorghum with antioxidant-rich edible flowers such as hibiscus and rose is a novel approach. This combination not only enhances the antioxidant capacity and sensory appeal of the product but also offers a new direction for edible packaging research, aligning with consumer preferences for natural, healthy, and sustainable food products.

There is growing interest in edible packaging and the use of biodegradable materials, research combining roasted sorghum with edible flowers like hibiscus and rose to develop a biodegradable, antioxidant-rich bowl is limited. Previous studies have primarily focused on the individual applications of sorghum or edible flowers in food products, but their integration into a functional, sustainable packaging solution remains unexplored. This research addresses this gap by developing and standardizing an edible sorghum bowl, enhancing its functionality through roasting and incorporating antioxidant-rich edible flowers like hibiscus and rose in dry powder form. The study evaluates the functional properties, sensory appeal, and biodegradability of these sorghum bowls, assessing their suitability as sustainable alternatives to SUPs for serving snacks and savories through exposure tests, shelf life analyses, and degradation assessments. This innovative approach offers a unique contribution to sustainable packaging solutions and supports a shift toward environmentally responsible consumption practices.

## 2. Materials and Methods

### 2.1. Selection of Raw Materials

**Figure 1:**
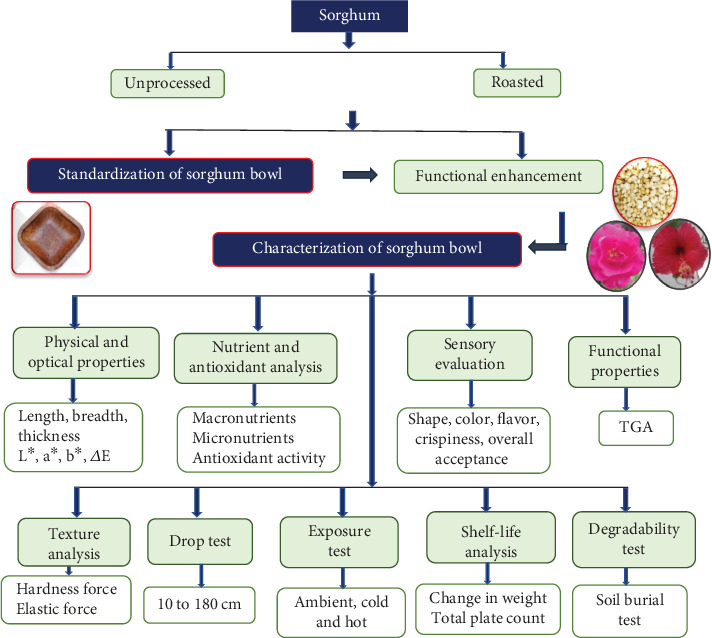
Schematic diagram of the development of plant-based edible bowl.

Premium quality white sorghum grains were procured from a grocery store in Coimbatore, Tamil Nadu. The grains were cleaned, sorted, dried, milled, and stored below 10% moisture content. Fully bloomed hibiscus and damask rose flowers in red and pink shades, were harvested from the Karur district of Tamil Nadu during the flowering season from January to March 2022. Hibiscus and damask rose were selected for their high antioxidant and functional properties among various edible flowers available. Solvents and chemicals for analysis were purchased from Sigma–Aldrich, India, from a chemical vendor in Coimbatore, Tamil Nadu. The flow of the study is given in Figure 1.

### 2.2. Processing of Raw Materials

Clean, dried white sorghum grains were dry roasted in an iron pan for 12 min at 90°C, till a few grains popped to improve the organoleptic properties and physical and functional qualities of the sorghum bowl [16], and finely ground (rice and millet milling machine, domestic seller, Coimbatore, Tamil Nadu, India) into flour to standardize the roasted sorghum bowl. Petals from damask rose and hibiscus were separated, shade dried, finely powdered, and kept in an airtight container with less than 10% moisture for value addition in the standardized unprocessed and roasted sorghum bowl. Before fabricating and standardizing edible bowls, the technological and functional characteristics of raw and roasted sorghum grains [27] and rose and hibiscus edible flower powder [28] were examined.

### 2.3. Standardization of Sorghum Bowls With Functional Enhancement

**Figure 2:**
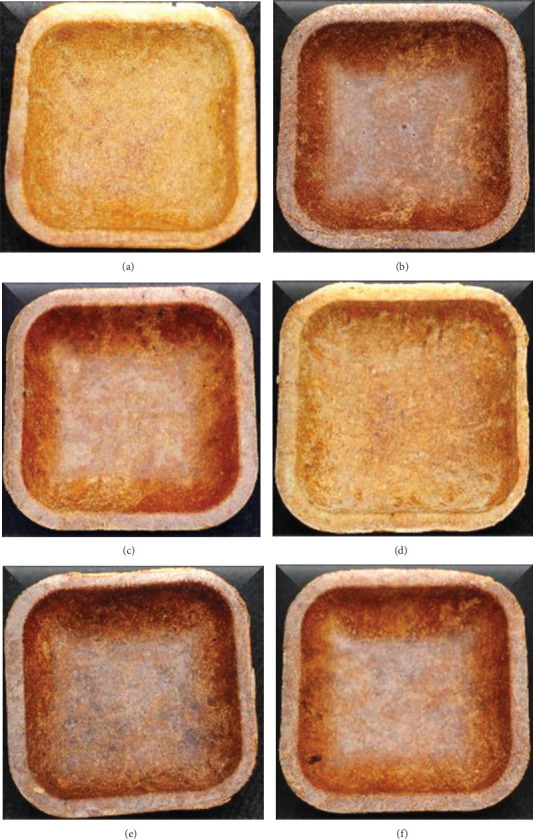
Standardized functionally enhanced sorghum bowls. (a) SUB, (b) SUHB, (c) SURB, (d) SRB, (e) SRHB, and (f) SRRB. SUB and SRB, sorghum unprocessed and roasted bowl; SUHB and SRHB, sorghum unprocessed and roasted hibiscus flower powder–enhanced bowl; SURB and SRRB, sorghum unprocessed and roasted rose flower powder–enhanced bowl.

Seven distinct compositions for sorghum bowls were tested, utilizing both roasted and unprocessed sorghum flour. The sorghum bowl was standardized with 14–16 g of sorghum flour, 5–6 g of wheat flour for binding, 2.5 g of jaggery for flavor, and 10 to 15 mL of water. The propositions were taken in a mixing bowl, kneaded into a soft dough, and then baked in a four-inch square-shaped unique mold at 80°C for 7 min at 1500 psi using baking and hydraulic pressure principles. Three grams of selected flower powder, such as hibiscus and rose, was added to enhance the functionality of both the unprocessed and roasted sorghum bowls. The standardized sorghum bowls were coded as sorghum unprocessed bowl (SUB), sorghum unprocessed hibiscus-incorporated bowl (SUHB), and sorghum unprocessed rose-incorporated bowl (SURB), whereas SRB stands for sorghum roasted bowl, SRHB stands for sorghum roasted hibiscus-incorporated bowl, and SRRB stands for sorghum roasted rose-incorporated bowl, as shown in Figure 2.

#### 2.3.1. Physical and Optical Properties of the Functionally Enhanced Sorghum Bowls

A vernier caliper was utilized to measure the height, width, and thickness of the standardized bowls. The average weight was determined using a digital laboratory scale weighing balance (Saffron). Optical properties of the standardized bowls were assessed using a Laboratory Scale Food Colorimeter, which provided varying values of *L*^∗^, *a*^∗^, *b*^∗^, and Δ*E*. *L*^∗^, ranging from 0 to 100, denotes lighter to darker shades, while positive and negative values of *b*^∗^ signify yellowness and blueness, respectively. Positive values of *a*^∗^ indicate redness, while negative values indicate greenness. Δ*E* represents the difference between chroma and hue [29]. The optical property was evaluated on Day 1 and Day 120 by storing it in an airtight container to analyze the color and hue changes of the sorghum bowl during storage.

### 2.4. Nutritional Analysis of the Functionally Enhanced Sorghum Bowls

Moisture and ash content were assessed by Association of Official Analytical Collaboration (AOAC) 930.15 and AOAC 942.05 standards. Carbohydrates, protein, fat, and crude fiber were analyzed by the anthrone method, AOAC 2001.11, AOAC 2003.05, and AOAC 97.10, respectively. Ascorbic acid assay, the Folin–Ciocalteu method, titration against potassium permanganate (KMnO_4_), and 1-amino-2-napthol-4-sulphonic acid (ANSA) were employed to quantify vitamin C, iron, calcium, and phosphorus [30, 31].

### 2.5. Phytochemical Screening of the Functionally Enhanced Sorghum Bowls

Screening of alkaloids, amino acids, anthocyanins, carbohydrates, flavonoids, glycosides, phenol, phytic acid, saponin, tannin, and terpenoids was assessed in aqueous, ethanol, chloroform, and acetone extracts to analyze the presence of phytochemicals [32].

### 2.6. Antioxidant Activity of the Functionally Enhanced Sorghum Bowls

1,1-Diphenyl-2-picrylhydrazyl (DPPH) scavenging activity assay was used to determine the antioxidant activity of a functionally enhanced sorghum bowl. IC50 values were calculated using linear regression. Strong antioxidant activity is defined as having an IC50 value between 10 and 50 mg/mL, intermediate antioxidant activity between 50 and 100 mg/mL, and poor antioxidant activity over 100 mg/mL [33].

### 2.7. Antinutritional Factors of the Functionally Enhanced Sorghum Bowls

The Folin–Denis spectrophotometric technique was used to estimate the tannin content in functionally enhanced sorghum bowl samples, and the Folin–Ciocalteu reagent was used to examine the total phenolic content [34]. A spectrophotometer method against iron(III) nitrate (Fe(NO_3_)_3_) equivalent was used to determine the phytic acid content [35].

### 2.8. Sensory Evaluation of the Functionally Enhanced Sorghum Bowls

A panel consisting of 30 semitrained female panelists assessed the sensory characteristics of the sorghum bowl. Traits like shape, color, taste, flavor, crispiness, and overall acceptance were measured using a nine-point hedonic scale (1 being *disliked extremely* to 9 being *like extremely*) at 11:00 a.m. Sensory changes were observed on day 1 and day 120 by storing in an airtight container as a quality indicator and significant likelihood indicator of the consumers [36].

### 2.9. Functional Properties of the Functionally Enhanced Sorghum Bowls

The peaks from the Fourier transform infrared spectrophotometer (FTIR) (Shimadzu MIRacle10) were used to identify the functional groups of the standardized sorghum bowls. The spectra of 0.5 g of sample were obtained, at 16 runs per scan, and the peaks were identified between 4000 and 450 cm^−1^ wavenumbers [16, 36]. Thermogravimetric analyzer (TGA) (EXSTAR/C300) was used to identify the thermal properties and to calculate the percentage of thermal deterioration as the bowl endures certain physiochemical changes in response to the controlled rise in temperature. The sample was heated in an alumina pan from 20°C to 1000°C at 20°C increase per minute [36].

### 2.10. Texture Analyzer of the Functionally Enhanced Sorghum Bowls

Hardness force, elastic force, and break force were measured by a texture analyzer **(**Shimadzu EZ-XS texture analyzer). Measurements were taken at a speed of 1 mm per second until the bowl broke into fragments at three bend points [36].

### 2.11. Exposure Properties of the Functionally Enhanced Sorghum Bowls

Bowls with snacks and savories were subjected to exposure for an hour in ambient, high, and low temperatures at 36°C, 80°C, and −5°C, and the changes were observed to identify the thermal retaining property of the standardized sorghum bowls for 1 h. Fresh dry and crunchy snacks, sweets with syrups, and hot soup or curry were taken for exposure test and observed for 90 min in a controlled room environment at 36°C.

### 2.12. Drop Resistance of the Functionally Enhanced Sorghum Bowls

ASTM D5276 standards were used to measure drop resistance that serves as a proxy for free fall impact. The drop resistance of standardized sorghum bowls was tested by dropping from a height of 50 to 180 cm, and the resulting changes were recorded [37].

### 2.13. Cytotoxicity Analysis of the Functionally Enhanced Sorghum Bowls

Brine shrimp lethality assay of functionally enhanced sorghum bowl was done at five different concentrations (100, 250, 500, 1000, and 1500 mg/mL) against brine solution as blank and potassium dichromate as a positive control for 24 h, and the lethality was observed at 1, 2, 4, 6, and 24 h. Thirty brine shrimps after 24 h of hatching were introduced in 25 mL of the sample containing brine solution to observe the mortality and percentage of lethality calculated (Equation (1)) [32].

(1) $$\begin{array}{c} \%\text{of mortality}=\frac{\text{Number of brine shrimp mortal}}{\text{Number of brine shrimp introduced}}\times 100 \end{array}$$

### 2.14. Shelf Life Evaluation of the Functionally Enhanced Sorghum Bowls

The total plate count (TPC) technique was used to examine the microbial load for 120 days at 30-day intervals [38]. Another aspect to consider in measuring shelf life is an increase in change and moisture percentage which was assessed by storing the bowls in air-tight containers at room temperature (30°C with 50% humidity) for 120 days to track the absorption of environmental moisture that affects the longevity of standardized edible sorghum bowls over time.

### 2.15. Degradability of the Functionally Enhanced Sorghum Bowls

Wet sandy topsoil from star garden, Avinashilingam Institute, Coimbatore, Tamil Nadu, India, was collected and a piece of bowl weighing 2 g was buried in the soil containing a beaker at a depth of 10 cm and tested till complete degradation in triplicates. The samples were removed from the soil on the third, sixth, and ninth day and dried in a hot air oven at 70°C for 24 h. The weight change and percentage of degradation (Equation (2)) were measured by the modified procedure [39].

(2) $$\begin{array}{c} \%\text{of degradation}=\frac{\text{Final weight}–\text{Initial weight of the sorghum bowl taken}}{\text{Initial weight }}\times 100 \end{array}$$

### 2.16. Statistical Analysis

One-way ANOVA followed by Duncan's post hoc test was conducted using Statistical Package for the Social Sciences (SPSS) software to determine significant differences among unprocessed, roasted, and functionally enhanced sorghum bowls at a significance level of 0.05 and a 95% confidence level. Graphs and figures were plotted through Origin 2024 (OriginPro Learning Edition) and Microsoft (MS) Excel.

## 3. Results/Discussion

### 3.1. Physical and Optical Properties of the Functionally Enhanced Sorghum Bowls

Standardized bowls were 10.2 cm × 10.2 cm square shaped with curved edges at the upper surface and the bottom/lower surface is 8.9 cm × 8.9 cm which shows 0.65 cm broad upper edges with an average thickness of 0.3 cm. The average weight of the SUB, SUHB, SURB, SRB, SRHB, and SRRB were 19.42, 18.95, 18.47, 19.09, 18.65, and 18.43 g, respectively.  Table 1 presents the optical properties of functionally enhanced sorghum bowls, specifically measuring lightness (*L*^∗^), redness (+*a*^∗^), yellowness (+*b*^∗^), and total color difference (Δ*E*) on Day 1 and Day 120. On Day 1, the SUB showed the highest lightness (11.62), while the SUHB had the lowest (7.45). The redness (*a*^∗^) values ranged from 3.48 (SRHB) to 4.79 (SRRB), indicating variations in color intensity. Yellowness (*b*^∗^) was highest in SUB (5.00) and lowest in both hibiscus-incorporated bowls (SRHB and SUHB, 1.71). The total color difference (Δ*E*) showed that SRB (4.51) and SUB (4.35) had the most significant variation, while SRRB (1.88) had the least. Similarly, on Day 120, all bowls demonstrated changes in their optical properties. Lightness (*L*^∗^) increased for most samples, with SUB (13.62) and SRB (12.11) showing the highest values, indicating possible lightening over time. Redness (*a*^∗^) ranged from 3.98 (SRB) to 6.11 (SURB), with roasted samples showing distinct differences compared to their unprocessed counterparts. Yellowness (*b*^∗^) also increased, especially in SUB (5.67) and SRB (5.45), suggesting that these samples became more yellow over time. The total color difference (Δ*E*) was most pronounced in SUB (5.68) and SRB (5.18), while SRRB had the smallest difference (2.16), indicating more stable color retention. Comparing Days 1 and 120, the flower-enhanced and roasted bowls (SRB, SRHB, and SRRB) showed minimal differences in optical properties compared to unprocessed sorghum, suggesting better stability over time. The *F*-values (*p* < 0.05) for optical properties indicate that differences between the samples were statistically significant, highlighting the impact of formulation and processing on color stability and visual appeal of the sorghum bowls. The observed changes in color can be attributed to chemical reactions and transformations occurring in cereal-based products under elevated temperatures. Processes such as the Maillard reaction, moisture absorption, and starch gelatinization contribute to the darkening effect. Additionally, the pronounced red, yellow, and total color variations are likely due to pigment leaching and the oxidation of polyphenols, which are characteristic of heat treatment processes [16, 29, 40]. The findings for flower powder-enhanced bowls align closely with biscuits containing 10%–15% rose calyx powder, which exhibit a distinct reddish tint and a slight reduction in brightness as rose content increases. Bioactive-rich flowers, especially those high in anthocyanins, serve as natural colorants that intensify red and yellow hues in edible cutlery [41, 42].

### 3.2. Nutritional Analysis of the Functionally Enhanced Sorghum Bowls

The nutritional analysis of functionally enhanced sorghum bowls is depicted in Table 2. The moisture content of SUB, SRB, and functional enhancement of edible bowls showed similar results between 6.2 and 7.6 percent. The ash content of functionally enhanced roasted and SUBs does not show any significant difference. SUB and SRB showed significant differences, and functional enhancement did not show any significant differences in the carbohydrate content. SRB, SRHB, and SRRB showed higher protein content than SUB, SUHB, and SURB. The fat content of the sorghum bowl exhibited a significant difference between unprocessed and roasted bowls, and the functional enhancement of flower powder did not exhibit a significant difference. Fiber content is increased by both roasting and incorporation of hibiscus and rose flowers and exhibited significant differences. Sorghum, hibiscus, and rose are poor sources of vitamin C as the SUB and SRB showed significant differences and functional enhancement has not shown a significant difference. The iron content of the incorporation of flowers showed a significant difference. The calcium and phosphorous content of SURB and SRRB is highest with 6.83, 53.49, and 6.75, 52.6 mg, respectively, among the other sorghum bowls. Nutritional analysis of the sorghum bowls shows that flower powder incorporation significantly increases the micronutrient content and roasting decreases the fat content. Macronutrients like carbohydrates, protein, and fiber and micronutrients like calcium and phosphorous were increased whereas fat, iron, and vitamin C were decreased in roasted sorghum bowls. The results are consistent with earlier research, and sorghum is a rich source of dietary fiber, minerals, and antioxidants and is suitable for gluten-free product formulations [34]. Ash, carbohydrate, iron, calcium, and protein content increased while, fat, and dietary fiber content decreased during the roasting of sorghum due to alter in the chemical composition [43, 44]. Multimillet edible bowls provide a higher content of dietary fiber and protein while delivering lower levels of calories, fat, and moisture [10]. The addition of roselle calyx powder biscuits had a significant influence on protein, ash, fat, or carbohydrate content [41].

### 3.3. Phytochemical Screening of the Functionally Enhanced Sorghum Bowls

**Figure 3:**
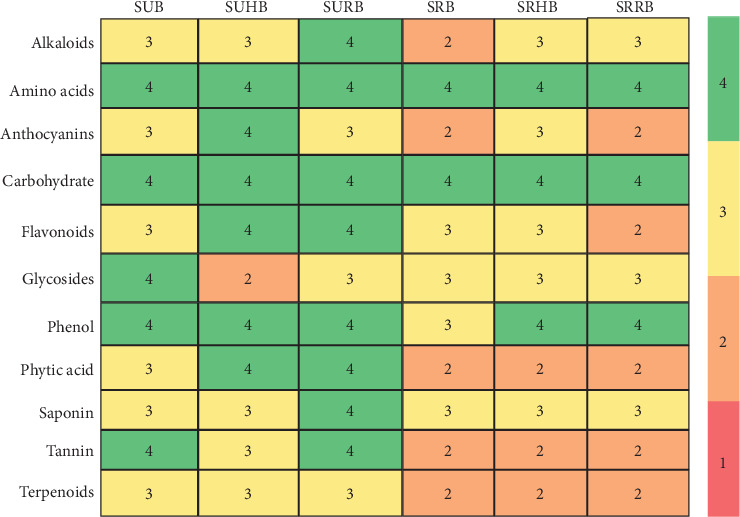
Heatmap for phytochemical screening of functionally enhanced sorghum bowl in aqueous, ethanol, chloroform, and acetone extracts. SUB & SRB, sorghum unprocessed and roasted bowl; SUHB & SRHB, sorghum unprocessed and roasted hibiscus flower powder–enhanced bowl; SURB & SRRB, sorghum unprocessed and roasted rose flower powder–enhanced bowl.

The presence of alkaloids, amino acids, anthocyanins, carbohydrates, flavonoids, glycosides, phenol, phytic acid, saponin, tannin, and terpenoids in aqueous, ethanol, chloroform, and acetone extracts in SUB, SUHB, SURB, SRB, SRHB, and SRRB were analysis and depicted (Figure 3). Alkaloids were not found in the aqueous medium except SURB and not in the ethanol medium in SRB. Amino acids and carbohydrates were present in all four mediums in all six bowls. Anthocyanins exhibited presence in chloroform and acetone medium than aqueous and ethanol extract. Flavonoids and glycosides were exhibited in ethanol, chloroform, and acetone medium. The intensified color changes observed for phytic acid, tannins, saponins, and terpenoids in unroasted samples (SUB, SUHB, and SURB) compared to roasted variants (SRB, SRHB, and SRRB) indicate that roasting effectively reduces phytochemicals and antinutritional factors in sorghum. Additionally, the greater color intensity of alkaloids, anthocyanins, and phenols in flower powder–enriched samples (SURB, SUHB, SRHB, and SRRB) compared to SUB and SRB confirms that flower powder incorporation enhances the presence of beneficial phytochemicals. These outcomes were in par with the earlier finds; sorghum exhibits the presence of steroids, glycosides, saponins, tannins, and alkaloids, and roasting decreases the presence of alkaloids, tannins, and phytic acid [45, 46]. The presence of anthocyanins, carotenoids, flavonol, and flavonols in rose and hibiscus flowers was exhibited through nuclear magnetic resonance (NMR) and chromatographic studies [47, 48].

### 3.4. Antioxidant Activity of the Functionally Enhanced Sorghum Bowl

**Figure 4:**
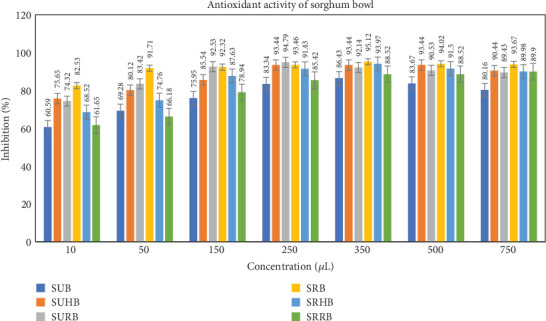
Antioxidant activity of standardized functionally enhanced sorghum bowls. SUB & SRB, sorghum unprocessed and roasted bowl; SUHB & SRHB, sorghum unprocessed and roasted hibiscus flower powder–enhanced bowl; SURB & SRRB, sorghum unprocessed and roasted rose flower powder–enhanced bowl.

The antioxidant activity of the sorghum bowl was evaluated in the aqueous extract at various concentrations of 10, 50, 150, 250, 350, 500, and 750 *μ*L, and the values are depicted in the graph (Figure 4). IC50 values were measured using linear regression to determine the efficacy of sorghum bowls in inhibiting radical scavenging activity. IC50 values of SUB, SUHB, and SURB showed 51.91, 47.74, and 39.87 mg/mL. In contrast, SRB, SRHB, and SRRB exhibited 72.99, 54.99, and 46.76 mg/mL revealing that sorghum bowls with functional enhancement of hibiscus and rose showed stronger radical scavenging activity than unprocessed and roasted sorghum bowls, and SUB exhibited stronger antioxidant activity than roasted sorghum bowl. These trends are consistent with earlier observations, unprocessed sorghum flour had higher DPPH radical scavenging activity than roasted sorghum flour as the Maillard reaction masked phenolic compounds [16]. Roasted sorghum grain had a much greater total phenolic count, total alkaloid content, and total flavonoid content than the raw sorghum grain, but the antioxidant activity was not affected indicating that other compounds enhance the antioxidant activity [48]. Fresh and spent rose flowers and hibiscus have potent antioxidant properties that prevent free radical-induced ailments [23, 26].

### 3.5. Antinutritional Factors of the Functionally Enhanced Sorghum Bowls

The antinutritional factors of the functionally enhanced bowls are presented in Table 3. The SUB, SUHB, and SURB varieties exhibited significantly higher levels of antinutritional factors compared to SRB, SRHB, and SRRB, suggesting that roasting sorghum effectively reduces total phenols, tannins, and phytic acid content. Adding dry flower powder notably increased the total phenolic content, although it had a lesser impact on tannin and phytic acid levels. Rose flower powder, in particular, contributed to a higher total phenolic content than hibiscus flower powder. Previous studies reported that unprocessed sorghum had a range of 1.67–0.16 mg/g of phytic acid, 1.58–12.97 mg/g of tannins, and 12.35–0.89 mg/g of total polyphenols, respectively [35]. High levels of antinutritional factors in food crops can negatively impact nutrient availability and digestion, making them unsuitable for human consumption without proper processing [49]. Preprocessing including roasting sorghum and other millets is effective in reducing the levels of phytic acid and tannins due to its heat-liability, thereby enhancing the nutritional quality of the food [13, 34, 45, 50]. Incorporating 15% rose calyx powder significantly boosted the phenolic content in biscuits, raising it from 3 mg gallic acid equivalent (GAE)/100 g to 76 mg GAE/100 g [41].

### 3.6. Sensory Evaluation of the Functionally Enhanced Sorghum Bowls

**Figure 5:**
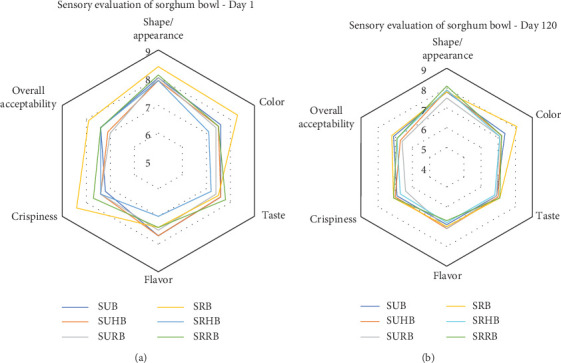
Sensory evaluation of the functionally enhanced sorghum bowls (a) at Day 1 and (b) at Day 120. SUB & SRB, sorghum unprocessed and roasted bowl; SUHB & SRHB, sorghum unprocessed and roasted hibiscus flower powder–enhanced bowl; SURB & SRRB, sorghum unprocessed and roasted rose flower powder–enhanced bowl.

Thirty semitrained panel members evaluated the sensory attributes of sorghum bowls using a nine-point hedonic scale and the results were portrayed (Figure 5) as a radar chart. The taste preference of the sorghum bowls was between 7.9 and 8.1 which falls under like very much category. The color of the sorghum bowls was above 7.1 which was liked moderately and the highest score (8.3) was in SRB, whereas SRRB (7.8) exhibited the highest taste pleasure and SUHB (6.6) resulted in the least taste preference. SURB showed the highest flavor preference and SUB showed the lowest flavor preference with a score of 6.7. The appearance of the sorghum bowls showed moderate to like very much preferences. Overall acceptability of SRB was higher followed by SRHB and SRRB with 7.9 and 7.4 scores. All the sensory attributes showed that roasted sorghum bowls showed a higher preference than unprocessed bowls as roasting eliminates the raw flavor and taste of sorghum. SRHB and SRRB showed higher preferences than SUHB and SURB. The sensory acceptance of jackfruit seed ice cream cones depends on the quantity of water and seed flour incorporation [51]. Roasting of millets enhances the sensory attributes and prolongs shelf life [44]. Biscuits with 5% rose calyx powder were rated higher than those with 10% or 15% powder, with 5% rose powder biscuits having the highest overall acceptability [41].

### 3.7. Functional Properties of the Functionally Enhanced Sorghum Bowls

#### 3.7.1. FTIR of the Functionally Enhanced Sorghum Bowls

**Figure 6:**
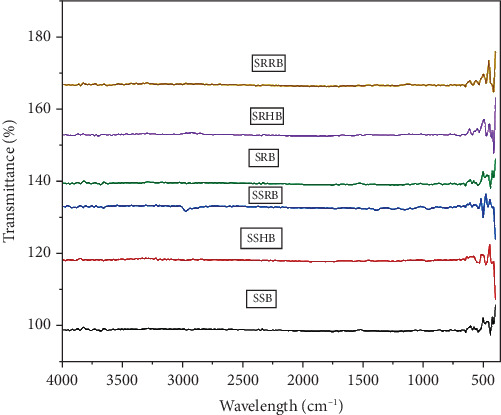
FTIR spectra of standardized plant-based edible bowls. SUB & SRB, sorghum unprocessed and roasted bowl; SUHB & SRHB, sorghum unprocessed and roasted hibiscus flower powder–enhanced bowl; SURB & SRRB, sorghum unprocessed and roasted rose flower powder–enhanced bowl.

FTIR peaks were measured to evaluate the presence of functional groups in standardized functionally enhanced sorghum bowls, and the graph was plotted (Figure 6) from 400 to 1000 cm^−1^ wavelength as the sorghum bowls showed peaks at this particular wavelength. Stretching peaks at 3826.77 cm^−1^ in SUB, SUHB, and SURB and weak stretching peaks at 3712.14 and 3914.12 cm^−1^ in SRB, 3703.33 cm^−1^ in SRHB, and 3849.92 cm^−1^ in SRRB showed breakdown of functional compounds due to baking, roasting, and thermal reactions and showed that similar results were observed in microwave roasted sorghum [16]. Asymmetrical starching peaks at 2978.09 revealed the presence of methylene (CH_2_) group of aliphatic chain compounds that were not present in SRB, SRHB, and SRRB due to roasting. Stretching peaks between 1110 and 990 showed the presence of C-O and C-C groups that reveal the presence of starch and similar results were observed in sorghum grain [52]. Stretching peaks observed at 725.23, 752.02, 754.48, 1149.57, and 1381.03 cm^−1^ in SUB, SUHB, and SURB indicate the presence of phenols; phenolic acids and flavonoids with the stretching given by C–C, C–H, and O–H deformations showed the presence of starch, and similar peaks was confirmed in dry heat treatment of sorghum [53]. The strong vibrational stretching peaks between 400 and 800 cm^−1^ showed the presence of amylose and amylopectin, and intensive peaks were exhibited in SUBs than roasted sorghum bowls. The roasted sorghum flour exhibited different peaks as compared to unprocessed flour due to changes in carbohydrate, protein, lipids, and phenolic compounds [16].

#### 3.7.2. TGA of the Functionally Enhanced Sorghum Bowls

**Figure 7:**
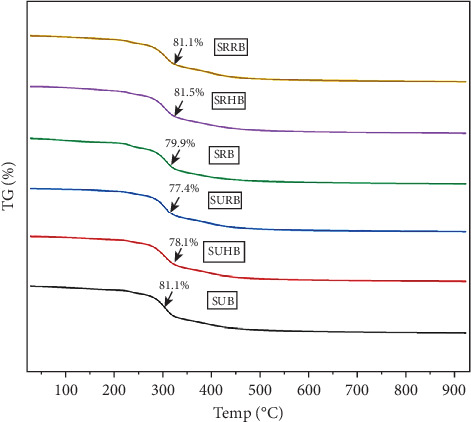
TG analysis of standardized edible bowls. SUB & SRB, sorghum unprocessed and roasted bowl; SUHB & SRHB, sorghum unprocessed and roasted hibiscus flower powder–enhanced bowl; SURB & SRRB, sorghum unprocessed and roasted rose flower powder–enhanced bowl.

Eight to ten milligrams of sorghum bowls was taken for thermal degradation analysis from 0°C to 1000°C, and the TGA peak is exhibited (Figure 7); at 150°C, SUB, SUHB, and SURB lost 11.9%, 5.3%, and 4.4%, whereas 6.67%, 8.39%, and 12.7% lost in SRB, SRHB, and SRRB due to moisture elimination. At 800°C, 67%–71.7% in SUB, SUHB, and SURB and 62.7%– 65.7% of total mass degradation occurred due to protein, carbohydrate, and lipids. At 1000°C, SUB, SUHB, and SURB experienced total weight losses of 81.1%, 78.1%, and 77.4%, leaving residue masses of 1.6, 1.82, and 1.79 mg, and SRB, SRHB, and SRRB exhibited thermal degradations of 79.9%, 81.5%, and 80.2%, leaving residues of 1.73, 1.27, and 2 mg including inorganic components and functional compounds. The derivative thermogravimetry (DTG) curve of the six samples showed a sharp decline at 317°C, exhibiting the fastest thermal decomposition. The initial step involves moisture dehydration from 25°C to 200°C, and protein and carbohydrate breakdown at 200°C–350°C due to depolymerization, decarboxylation, and cracking reactions. Lipid breaks from 350°C to 550°C, and other organic components degrade at 550°C–800°C [54].

### 3.8. Texture Analyzer of the Functionally Enhanced Sorghum Bowls

The hardness, break force, and elastic force were tabulated in Table 4. Hardness force and break force of SUB and SRB showed significant differences and functional enhancement also showed significant differences with a *p* value less than 5%. The highest force required to completely break was exhibited in SUHB, and the lowest resulted in SRHB. Functional enhancement of flowers in unprocessed bowls increased the hardness force, and in roasted sorghum bowls, flower enhancement decreased the hardness force. The elastic force in SUHB and SRB were similar with 373.87 and 373.16 N/mm^2^. The lowest elastic force was observed in SRRB (99.75 N/mm^2^), and all the bowls exhibited significantly different elastic forces. These findings align with previous studies, showing that the hardness of unprocessed sorghum bread increased from 13.34–21.09 to 19.20–50.31 due to starch integrity loss and a weakened gluten–starch network. Meanwhile, springiness decreased from 1.02–0.66 to 1.02–0.83, as better interactions between gelatinized starch and gluten improved dough elasticity, creating a cohesive sponge structure upon heating [55]. Similar studies show that the hardness force was 8.28–21.66 N and crispiness was from 0.92 to 2.41 force drops/mm, which were studied in the jackfruit seed flour–based waffle ice cream cones [51]. The texture properties of biobased edible bowls increased with increasing concentration of spent coffee grain bowls [39].

### 3.9. Exposure Test of the Functionally Enhanced Sorghum Bowls

**Figure 8:**
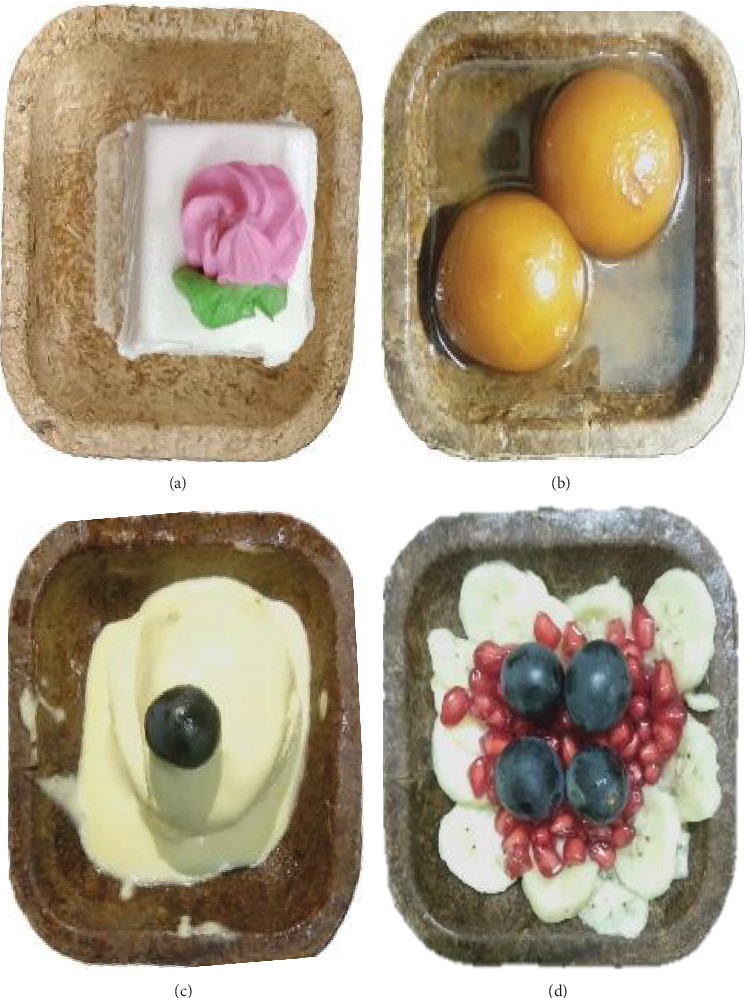
Exposure test of sorghum bowl: (a) cake, (b) sweet with sugar syrup, (c) ice cream, and (d) fruit bowl.

An exposure test was experimented with by exposing the bowls to 36°C, 80°C, and −5°C representing ambient, hot, and cold temperatures (Figure 8). Snacks and savories like boiled pulses, fruit salads, crunchy foods (vada, pani poori, and puffs), sweets (cakes), sweets with syrups (Gulab Jamun and rasa malai), ice cream, and side dishes (curries) were tested. Dry snacks and savories exhibited no effects, and wet snacks, savories, soup, and curries did not absorb for up to 60 min without change to their structural integrity; the inner side of the bowl started to absorb the sugar syrup or liquid food items after 90 min. Hot soups tend to absorb in the inner side after exposure for 75 min. The exposure test of the functionally enhanced edible sorghum bowls proved its suitability to serve snacks and savories in hospitality, entertainment, and service industries, and the functional enhancement of edible flowers did not affect the nature of the bowl. The jackfruit seed flour ice cream cones permeated to the outside of the cone within 40 min [51]. The sorghum edible cutlery was completely soggy within 30 min when exposed to hot soup [2]. Biobased edible bowls with spent coffee grain become soggy within 30 min of exposure to water [39]. Multimillet bowls exhibit reduced water absorption and a firmer, thicker surface texture, enhancing their durability and structural integrity [10].

### 3.10. Drop Resistance of the Functionally Enhanced Sorghum Bowls

**Figure 9:**
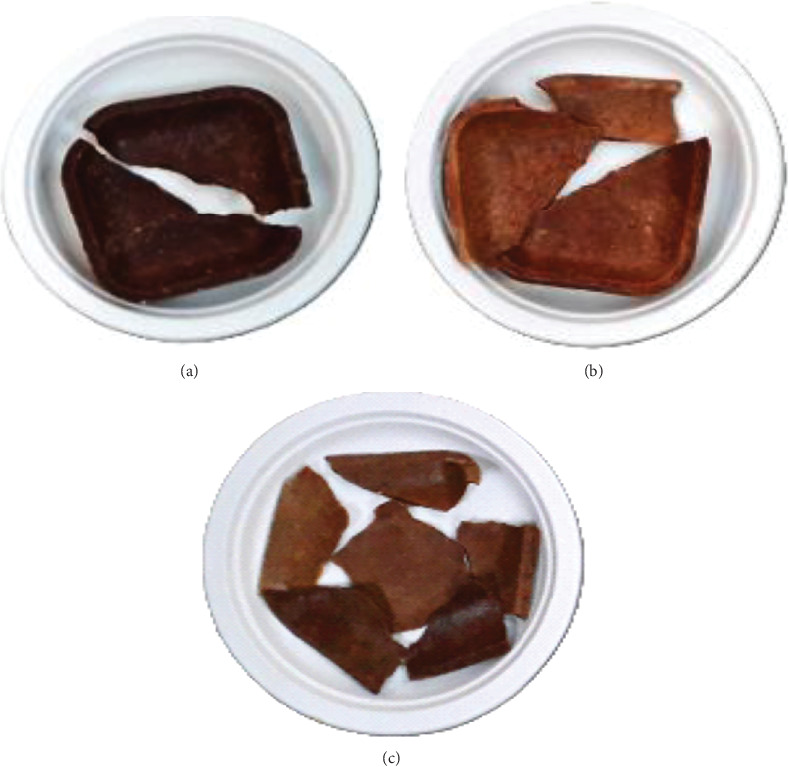
Drop test of sorghum bowls: (a) SUHB at 90 cm, (b) SUB at 110 cm, and (c) SRRB at 170 cm.

A drop test was done to analyze the impact of accidental falls during serving and handling by dropping from a height of 50–180 cm (Figure 9). No alterations or damage were noticed when SUB, SUHB, SURB, SRB, SRHB, and SRRB were dropped from a height of 50 to 90 cm. The first crack was found at 110 cm in SUB, SUHB, and SURB and at 120 cm in SRB, SRHB, and SRRB, respectively. At 150 cm, functionally enhanced SUHB, SURB, SRHB, and SRRB sorghum bowls were completely broken, and at 170 and 180 cm, SUB and SRB were completely broken, respectively. The free fall impact of sorghum bowls showed that the functionally enhanced bowls were broken earlier than the SRB and SUB.

### 3.11. Cytotoxicity Analysis of the Functionally Enhanced Sorghum Bowls

The brine shrimp lethality assay was conducted to evaluate the cytotoxicity of SUB, SUHB, SURB, SRB, SRHB, and SRRB over 24 h (Table 5). The results indicated minimal cytotoxicity across all tested samples. At concentrations of 100 and 500 mg/mL, all brine shrimp remained alive after 24 h for all the samples, indicating no significant lethality. Even at a higher concentration of 1000 mg/mL, SRB showed no mortality, while SUB and the flower powder–incorporated sorghum bowls exhibited only a 3% mortality rate. When the concentration was increased to 1500 mg/mL, SRB showed a slight increase in mortality (3%), SUB had a 7% mortality rate, and SUHB, SURB, SRHB, and SRRB each exhibited 10% mortality. The brine shrimp lethality assay demonstrated a minimal cytotoxic effect on brine shrimp nauplii, with a trend of increasing mortality corresponding to higher concentrations. The mortality rate was below 7% across all six sorghum bowls, making it impossible to calculate the LC50 value for concentrations ranging from 100 to 1500 *μ*g/mL for the roasted and edible flower powder–enhanced samples, indicating low toxicity. The LC50 value in a brine shrimp assay for sorghum and hibiscus aqueous extract was reported to be between 3.7 and 4.6 *μ*g/mL [56]. A 16.66% mortality rate was observed at a concentration of 1000 *μ*g/mL in rose extracts [57]. The cytotoxicity of these plant extracts is primarily attributed to the presence of bioactive compounds such as alkaloids, flavonoids, and tannins [57, 58].

### 3.12. Shelf Life Evaluation of the Functionally Enhanced Sorghum Bowls

TPC and moisture absorption through weight change were absorbed as quality indicators for the storage period of 120 days in an airtight container at room temperature (Tables 6 and 7). The TPC analysis of the sorghum-based edible bowls reveals that microbial growth gradually increases over time but remains within acceptable safety limits (Table 6). In SUB, the TPC rises from 2 × 10^1^CFU/g on Day 30 to 13 × 10^1^CFU/g by Day 120, while SUHB starts higher at 4 × 10^1^CFU/g on Day 30, reaching 15 × 10^1^CFU/g by Day 120. Similarly, SURB shows a gradual increase from 2 × 10^1^CFU/g to 13 × 10^1^CFU/g over 120 days. This higher microbial load suggests that the addition of hibiscus and rose powder may contribute to initial microbial counts or promote growth. In contrast, the roasted bowls demonstrate significantly better microbial stability. SRB maintains a TPC of 0 × 10^1^CFU/g from Day 30 to Day 90, with a minimal increase to 1 × 10^1^CFU/g by Day 120, suggesting that roasting has a sterilizing effect or creates a less favorable environment for microbial growth. SRHB and SRRB also show lower TPCs compared to their unroasted counterparts, with SRHB rising from 2 × 10^1^CFU/g to 11 × 10^1^CFU/g and SRRB from 3 × 10^1^CFU/g to 10 × 10^1^CFU/g by Day 120. The results highlight that roasting not only reduces initial microbial loads but also slows microbial proliferation, likely due to reduced moisture content and the elimination of certain microorganisms during roasting. While the addition of hibiscus and rose powders slightly increases the microbial load, the overall TPC in roasted bowls remains lower, underscoring the effectiveness of roasting in enhancing microbial control. All sorghum bowls demonstrate relatively low TPCs up to 120 days, indicating their microbiological stability for at least 4 months under controlled conditions. Among all samples, the SRB shows the most promising results, suggesting its potential for extended shelf life and making it an ideal choice for greener food serving cutlery. The sorghum *mahewu* products showed TPCs ranging from 7.914 to 8.978 log10 (CFU/mL) [40].

The sorghum-based edible bowls' moisture content and weight gain (SUB, SUHB, SURB, SRB, SRHB, and SRRB) provide valuable insights into their stability and potential shelf life (Table 7). Higher moisture content increases the risk of microbial spoilage and compromises product stability, while minimal weight gain is desirable to maintain texture and integrity. SUB shows a steady increase in moisture content from 6.76% on Day 1 to 8.89% by Day 120, with a corresponding weight rise from 19.42 to 19.84 g. This indicates moisture absorption from the environment over time, potentially compromising structural integrity and increasing microbial growth risk. SUHB begins with a higher moisture content of 7.6%, reaching 10.08% by Day 120, and a weight increase from 18.95 to 19.43 g. Similarly, SURB shows moisture content rising from 7.41% to 9.97% and weight from 18.47 to 18.95 g. The higher moisture retention compared to SUB suggests that the hibiscus or rose powder contributes to increased water absorption, affecting texture and stability. In contrast, SRB displays the least increase in moisture content and weight, from 6.26% to 7.23% and 19.01 to 19.20 g, respectively. This minimal increase suggests that roasting effectively reduces water absorption, enhancing shelf life and maintaining the bowl's texture and integrity longer than its unroasted counterpart. SRHB, with moisture content increasing from 6.5% to 8.09% and weight from 18.65 to 18.95 g, and SRRB, with moisture content rising from 6.76% to 8.54% and weight from 18.43 g to 18.73 g, show a significantly lower increase than SUHB, highlighting roasting's role in reducing moisture uptake and preserving structural integrity and confirming its potential to extend shelf life. Functional enhancement of edible flowers showed increased colony count and increased weight which can be overcome by freeze or cabinet drying. The dried rose extract has the lowest inhibitory concentration for pathogens, *E. coli*, *B. cereus*, and *Staphylococcus epidermidis* [24]. Roasting millets decreases the moisture content and prolongs shelf life [44].

### 3.13. Degradability of the Functionally Enhanced Sorghum Bowls

**Figure 10:**
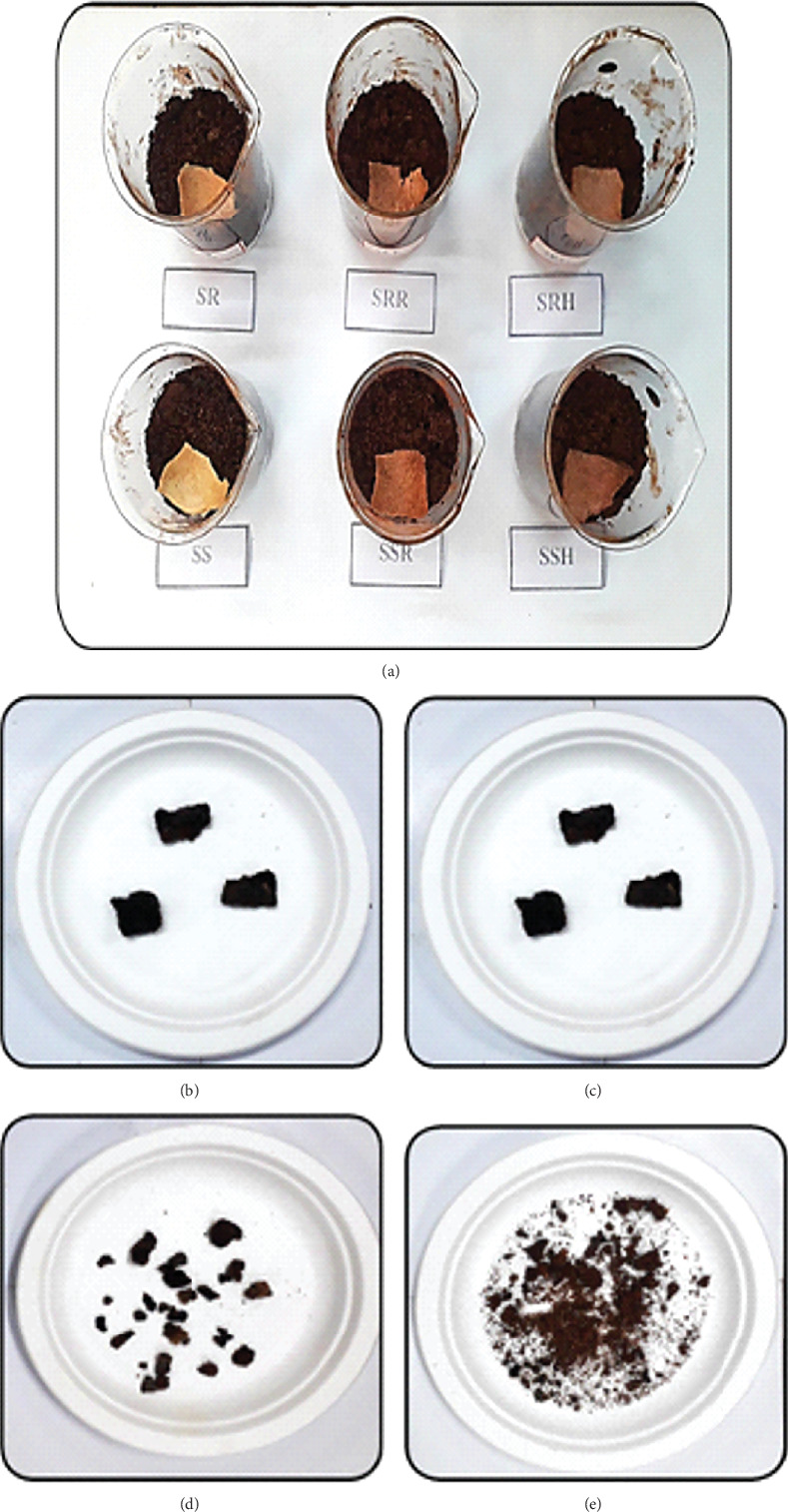
Soil burial test of edible bowls. (a) Sample buried in soil containing beak. (b) Day 3. (c) Day 6. (d) Day 9. (e) Day 12.

The biodegradability of 2 g of functionally enriched sorghum bowl in a soil environment was assessed by burying it at a depth of 10 cm in moist topsoil. The percentage degradation was subsequently monitored every 3 days (Figure 10). The initial weights of all bowl samples ranged from 2.01 to 2.17 g. By Day 3, all samples experienced a noticeable weight reduction, ranging from 1.69 g (SURB) to 1.91 g (SRRB), indicating the beginning of the degradation process with a degradation percentage of 19.5–29. On Day 6, further degradation was evident, with weights dropping significantly, from 1.01 g (SURB and SRHB) to 1.08 g (SUB). The weight reduction trend continued over the next days, with Day 9 showing weights between 0.47 g (SRRB) and 0.71 g (SUB), suggesting a substantial breakdown of the material. On Day 12, the weights of the samples were drastically reduced to a range of 0.09 g (SRRB) to 0.17 g (SURB). This reduction signifies that all sorghum bowls underwent extensive degradation within 12 days. The percentage degradation from Day 0 to Day 12 reveals high biodegradability rates: SUB degraded by approximately 95.0%, SUHB by 94.7%, SURB by 91.7%, SRB by 94.4%, SRHB by 94.0%, and SRRB by 95.8%. Among these, SRRB exhibited the highest degradation percentage, suggesting that it is the most biodegradable under soil conditions, followed closely by SUB and SRB. The result indicates that all bowls possess significant biodegradability, proving they are suitable for environmentally friendly applications. The sorghum edible spoon initially absorbed water, and 33.3% of degradation was obtained in 24 h [48]; biobased edible bowls with spent coffee grain degraded almost 30% in 10 weeks [39], and the multimillet edible bowl degraded in 42 days [10].

In conclusion, this research highlights the potential of sorghum-based edible bowls, particularly when enhanced through roasting and the addition of hibiscus and rose flower powders. The development of these ecofriendly, edible bowls involved optimizing the proportions of sorghum flour (70%–80%), wheat flour (15%–20%), and jaggery (5%) with 15% flower powder for functional benefits. The finalized formulation was thoroughly validated through optical property analysis, proximate composition, antinutritional and antioxidant activity assays, mechanical testing, cytotoxicity evaluation, shelf life assessments, and biodegradability studies.

The optical properties of roasted sorghum bowls (SRB, SRHB, SRRB) were similar to unprocessed ones, suggesting stability over time. Statistical analysis (*p* < 0.05) revealed that formulation and processing significantly impacted color stability. Proximate analysis indicated that flower powder enrichment boosted micronutrients, while roasting reduced fat content. Carbohydrates, protein, fiber, calcium, and phosphorus levels increased with roasting, whereas fat, iron, and vitamin C content decreased. Bowls containing hibiscus and rose powders demonstrated superior antioxidant activity compared to both unprocessed and roasted counterparts. Sensory evaluations favored roasted bowls, especially SRHB and SRRB.

Spectroscopic analysis confirmed the presence of starch and phenolic compounds, with stronger peaks in unprocessed bowls. Thermal analysis indicated significant weight loss at 1000°C, leaving residual inorganic and functional compounds. Functional enhancements improved hardness in unprocessed bowls but reduced it in roasted ones, with SRRB showing the lowest elasticity (99.75 N/mm^2^). Structurally, the bowls held up to 60 min with dry snacks and 75 min with hot soups, with sugar syrups starting to absorb after 90 min. Drop tests showed that enhanced bowls broke sooner than unprocessed or solely roasted versions, withstanding drops from 50 to 90 cm but breaking at 170–180 cm. The brine shrimp assay demonstrated low cytotoxicity, with increased mortality at higher concentrations. Roasting reduced initial microbial loads and delayed growth, thus extending shelf life. In biodegradability tests, all bowls decomposed fully within 12 days in wet topsoil.

These enhancements not only increase the bowls' nutritional value, antioxidant capacity, and sensory appeal but also improve their functional durability, allowing them to hold hot and cold foods for over an hour. Sorghum-based bowls present a sustainable alternative for the food, hospitality, and entertainment industries, potentially replacing SUPs. Their biodegradability supports waste reduction without requiring extra utensils or packaging. Suitable for all age groups, these bowls offer essential nutrients and add dietary variety. Commercializing these sorghum bowls could significantly reduce environmental pollution and contribute to global sustainability goals. Their adoption aligns with growing consumer demand for ecofriendly, health-conscious products and supports a shift toward a less plastic-dependent future. Further improvement in nutrition and antioxidant activity may be achieved by incorporating specific functional compounds and additional ingredients.

## Figures and Tables

**Table 1:** Optical properties of the functionally enhanced sorghum bowls.

**Optical property**	**L** ^∗^	**a** ^∗^	**b** ^∗^	Δ**E**
*At Day 1*				
SUB	11.62 ± 0.84^d^	4.17 ± 0.54^ab^	5 ± 0.94^c^	4.35 ± 0.52^b^
SUHB	7.45 ± 0.48^a^	3.8 ± 0.52^ab^	1.71 ± 0.03^a^	2.1 ± 0.51^a^
SURB	9.83 ± 0.4^c^	4.77 ± 0.41^b^	4.19 ± 0.05^c^	2.56 ± 0.16^a^
SRB	10.78 ± 0.4^d^	3.64 ± 0.66^a^	4.78 ± 0.66^c^	4.51 ± 0.82^b^
SRHB	7.94 ± 0.06^ab^	3.48 ± 0.57^a^	1.71 ± 0.03^a^	2.01 ± 0.58^a^
SRRB	8.48 ± 0.45^b^	4.79 ± 0.45^b^	3.06 ± 0.02^b^	1.88 ± 0.35^a^
*F* value	33.501⁣^∗^	3.14⁣^∗^	29.26⁣^∗^	15.422⁣^∗^

*At Day 120*				
SUB	13.62 ± 1.20^c^	5.17 ± 0.54^bc^	5.67 ± 0.53^c^	5.68 ± 1.10^c^
SUHB	8.45 ± 0.48^a^	4.8 ± 0.48^ab^	2.38 ± 0.57^a^	2.76 ± 0.59^ab^
SURB	10.83 ± 0.70^b^	6.11 ± 0.83^c^	4.85 ± 0.61^bc^	3.41 ± 0.41^b^
SRB	12.11 ± 0.75^b^	3.98 ± 0.69^a^	5.45 ± 0.49^c^	5.18 ± 0.36^c^
SRHB	8.61 ± 0.63^a^	4.97 ± 0.18^ab^	2.05 ± 0.60^a^	2.40 ± 0.64^ab^
SRRB	8.96 ± 0.05^a^	5.23 ± 0.28^bc^	3.99 ± 0.07^b^	2.16 ± 0.30^a^
*F* value	25.83⁣^∗^	4.73⁣^∗^	26.95⁣^∗^	16.67⁣^∗^

*Note:*Mean ± SD (*n* = 3): *p* value = 0.05. Values in superscript in each column denote the significant difference (*p* < 0.05). *L*^∗^: lightness; +*a*^∗^: redness; +*b*^∗^: yellowness; Δ*E*: total color difference.

Abbreviations: SUB & SRB, sorghum unprocessed and roasted bowl; SUHB & SRHB, sorghum unprocessed and roasted hibiscus flower powder–enhanced bowl; SURB & SRRB, sorghum unprocessed and roasted rose flower powder–enhanced bowl).

^*^Significance.

**Table 2:** Nutritional analysis of the functionally enhanced sorghum bowls.

**Nutritional analysis**	**SUB**	**SUHB**	**SURB**	**SRB**	**SRHB**	**SRRB**	**F** ** value (** **p** ** value)**
Moisture (%)	6.76 ± 0.45	7.6 ± 0.51	7.41 ± 0.52	6.26 ± 1.08	6.5 ± 0.49	6.76 ± 0.6	1.88⁣^∗∗^ (0.17)
Ash (%)	3.14 ± 0.02	3.37 ± 0.15	3.18 ± 0.03	3.27 ± 0.14	3.27 ± 0.05	3.24 ± 0.04	2.29 (0.11)⁣^∗∗^
Carbohydrate (g)	15.09 ± 0.02	15.31 ± 0.19	15.18 ± 0.04	15.35 ± 0.16	15.3 ± 0.02	15.25 ± 0.04	2.45 (0.09)⁣^∗∗^
Protein (g)	2.52 ± 0.02^a^	2.53 ± 0.02^a^	2.44 ± 0.09^a^	2.56 ± 0.13^a^	2.77 ± 0.06^b^	2.75 ± 0.07^b^	9.27 (0.001)⁣^∗^
Fat (g)	0.47 ± 0.02^bc^	0.51 ± 0.02^c^	0.41 ± 0.07^ab^	0.37 ± 0.05^a^	0.43 ± 0.02^ab^	0.44 ± 0.02^abc^	3.93 (0.02)⁣^∗^
Fiber (g)	2.52 ± 0.06^a^	2.63 ± 0.10^ab^	2.53 ± 0.09^a^	2.55 ± 0.07^a^	2.77 ± 0.05^c^	2.71 ± 0.02^bc^	5.76 (0.006)⁣^∗^
Vitamin C (mg)	0.10 ± 0.02^b^	0.12 ± 0.01^b^	0.10 ± 0.01^b^	0.04 ± 0.02^a^	0.07 ± 0.01^a^	0.05 ± 0.01^a^	11.36 (0.00)⁣^∗^
Iron (mg)	0.94 ± 0.04^b^	1.03 ± 0.08^b^	1.01 ± 0.05*b*	0.84 ± 0.06^a^	0.91 ± 0.06^b^	0.95 ± 0.02^b^	6.29 (0.004)⁣^∗^
Calcium (mg)	6.66 ± 0.01^a^	6.77 ± 0.02^bc^	6.83 ± 0.02^c^	6.75 ± 0.04^ab^	6.73 ± 0.07^ab^	6.75 ± 0.03^abc^	3.62 (0.03)⁣^∗^
Phosphorous (mg)	51.6 ± 0.98^a^	53.46 ± 0.14^c^	53.17 ± 0.07^bc^	51.94 ± 0.09^a^	52.76 ± 0.09^b^	52.6 ± 0.05^b^	13.84 (0.00)⁣^∗^

*Note:*Mean ± SD (*n* = 3), *p* value = 0.05. Values in superscript in each row denote the significant difference (*p* < 0.05).

Abbreviations: SUB & SRB, sorghum unprocessed and roasted bowl; SUHB & SRHB, sorghum unprocessed and roasted hibiscus flower powder–enhanced bowl; SURB & SRRB, sorghum unprocessed and roasted rose flower powder–enhanced bowl.

^*^Significance.

^**^No significance.

**Table 3:** Antinutritional factors of the functionally enhanced sorghum bowls.

**Antinutritional factors**	**SUB**	**SUHB**	**SURB**	**SRB**	**SRHB**	**SRRB**	**F** ** value**
Total phenolic content (mg/100 g GAE)	77.02 ± 2.28^b^	80.61 ± 1.02^c^	80.88 ± 1.54^c^	68.21 ± 1.10^a^	69.74 ± 0.39^a^	70.56 ± 0.60^a^	56.12 (0.00)⁣^∗^
Tannin (mg/100 g TAE)	8.33 ± 0.11^c^	8.61 ± 0.13^d^	8.75 ± 0.09^d^	3.63 ± 0.10^a^	3.77 ± 0.06^ab^	3.87 ± 0.06^c^	2089.31 (0.00)⁣^∗^
Phytic acid (mg/100 g)	28.89 ± 0.78^c^	29.95 ± 0.13^d^	30.11 ± 0.13^d^	27.25 ± 0.35^a^	28.14 ± 0.36^b^	29.21 ± 0.29^c^	21.60 (0.00)⁣^∗^

*Note:*Mean ± SD (*n* = 3), *p* value = 0.05. Values in superscript in each row denote the significant difference (*p* < 0.05).

Abbreviations: SUB & SRB, sorghum unprocessed and roasted bowl; SUHB & SRHB, sorghum unprocessed and roasted hibiscus flower powder–enhanced bowl; SURB & SRRB, sorghum unprocessed and roasted rose flower powder–enhanced bowl).

^*^Significance.

**Table 4:** Hardness, break force, and elastic force of functionally enhanced sorghum bowls.

**Texture analysis**	**SUB**	**SUHB**	**SURB**	**SRB**	**SRHB**	**SRRB**	**F** ** value**
Hardness and break force (*N*)	231.57 ± 7.31^ab^	458.4 ± 4.38^e^	238.09 ± 6.34^b^	310.94 ± 6.92^d^	227.72 ± 2.52^a^	282.27 ± 3.34^c^	783.262 (0.00)⁣^∗^
Elastic force (N/mm^2^)	158.7 ± 2.38^b^	373.87 ± 4.99^d^	229.39 ± 3.22^c^	373.16 ± 8.17^d^	216.49 ± 9.51^c^	99.75 ± 14.61^a^	546.71 (0.00)⁣^∗^

*Note:*Mean ± SD (*n* = 3), *p* value = 0.05. Values in superscript in each row denote the significant difference (*p* < 0.05).

Abbreviations: SUB & SRB, sorghum unprocessed and roasted bowl; SUHB & SRHB, sorghum unprocessed and roasted hibiscus flower powder–enhanced bowl; SURB & SRRB, sorghum unprocessed and roasted rose flower powder–enhanced bowl.

^*^Significance.

**Table 5:** Brine shrimp lethality assay of functionally enhanced sorghum bowls.

**Sorghum bowl**	**Mortality of brine shrimp (h)**	**Concentration (*μ*g/mL)**			
		**250**	**500**	**1000**	**1500**
SUB	2	0	0	0	0
	6	0	0	0	0
	24	0	0	1	2
	% mortality (24 h)	0	0	3	7

SUHB	2	0	0	0	0
	6	0	0	0	0
	24	0	0	1	3
	% mortality (24 h)	0	0	3	10

SURB	2	0	0	0	0
	6	0	0	0	0
	24	0	0	1	3
	% mortality (24 h)	0	0	3	10

SRB	2	0	0	0	0
	6	0	0	0	0
	24	0	0	0	1
	% mortality (24 h)	0	0	0	3

SRHB	2	0	0	0	0
	6	0	0	0	0
	24	0	0	1	2
	% mortality (24 h)	0	0	3	7

SRRB	2	0	0	0	0
	6	0	0	0	0
	24	0	0	1	2
	% mortality (24 h)	0	0	3	7

Abbreviations: SUB & SRB, sorghum unprocessed and roasted bowl; SUHB & SRHB, sorghum unprocessed and roasted hibiscus flower powder–enhanced bowl; SURB & SRRB, sorghum unprocessed and roasted rose flower powder–enhanced bowl).

**Table 6:** Total plate count of the functionally enhanced sorghum bowls.

**Edible bowls**	**Day 30**	**Day 60**	**Day 90**	**Day 120**
SUB	2 × 10^1^	4 × 10^1^	9 × 10^1^	13 × 10^1^
SUHB	4 × 10^1^	5 × 10^1^	11 × 10^1^	15 × 10^1^
SURB	2 × 10^1^	4 × 10^1^	10 × 10^1^	13 × 10^1^
SRB	0 × 10^1^	0 × 10^1^	0 × 10^1^	1 × 10^1^
SRHB	2 × 10^1^	4 × 10^1^	9 × 10^1^	11 × 10^1^
SRRB	3 × 10^1^	5 × 10^1^	9 × 10^1^	10 × 10^1^

Abbreviations: SUB & SRB, sorghum unprocessed and roasted bowl; SUHB & SRHB, sorghum unprocessed and roasted hibiscus flower powder–enhanced bowl; SURB & SRRB, sorghum unprocessed and roasted rose flower powder–enhanced bowl.

**Table 7:** Increase in weight and moisture content of the functionally enhanced sorghum bowls.

**Edible bowls**	**Day 1**		**Day 30**		**Day 60**		**Day 90**		**Day 120**	
	**Moisture (%)**	**Increase in weight (g/bowl)**	**Moisture (%)**	**Increase in weight (g/bowl)**	**Moisture (%)**	**Increase in weight (g/bowl)**	**Moisture (%)**	**Increase in weight (g/bowl)**	**Moisture (%)**	**Increase in weight (g/bowl)**
SUB	6.76	19.42	7.22	19.51	7.63	19.59	8.03	19.67	8.89	19.84
SUHB	7.6	18.95	7.91	19.01	8.21	19.07	9.25	19.27	10.08	19.43
SURB	7.41	18.47	8.11	18.60	8.70	18.71	9.28	18.82	9.97	18.95
SRB	6.26	19.01	6.62	19.08	6.93	19.14	7.13	19.18	7.23	19.20
SRHB	6.5	18.65	6.82	18.71	7.03	18.75	7.40	18.82	8.09	18.95
SRRB	6.76	18.43	7.08	18.49	7.24	18.52	7.90	18.61	8.54	18.73

Abbreviations: SUB & SRB, sorghum unprocessed and roasted bowl; SUHB & SRHB, sorghum unprocessed and roasted hibiscus flower powder–enhanced bowl; SURB & SRRB, sorghum unprocessed and roasted rose flower powder–enhanced bowl).

## Data Availability

Data is available on request from the authors.

## Supporting Information

Supporting Information Additional supporting information can be found online in the Supporting Information section.Figure S1 illustrates the nutritional analysis of the functionally enhanced sorghum bowl, as detailed in Table 2 and Section 3.2. The enhancement through roasting and the addition of flower powder significantly improves its nutritional value. Table S1 summarizes the phytochemical screening of the functionally enhanced sorghum bowl, discussed in Section 3.3 and Figure 3. The screening in aqueous, ethanol, chloroform, and acetone solvents reveals a strong presence of phytochemicals, indicating the enhanced bowl's robust phytochemical profile. Figure S2 depicts the antinutritional factors of the functionally enhanced sorghum bowl, elaborated in Table 3 and Section 3.5. Roasting significantly reduces antinutritional components such as total phenols, tannins, and phytic acid. Figure S3 presents a graph showing the toxicity analysis, total plate count, moisture content, and weight changes over 120 days, as part of the shelf life evaluation discussed in Sections 3.11 and 3.12 and detailed in Tables 5, 6, and 7. The toxicity analysis shows minimal toxicity, with fewer than three brine shrimp mortalities at the highest concentration (1500 *μ*g/mL). The results for moisture content, weight changes, and total plate count confirm that the sorghum bowl maintains a shelf life exceeding 120 days. Figure S4 provides a graphical representation of the soil burial test for the functionally enhanced sorghum bowl, discussed in Section 3.13 and Figure 10. The standardized sorghum bowl fully degrades in wet topsoil within 12 days.
