# Supplementary material for: Optimizing Edible Sorghum Bowls: Effects of Roasting and Edible Flower Powder Enhancement on Technological, Nutritional, Antioxidant, and Functional Properties
Source: Int J Food Sci. 2025 Jan 6;2025:1771084. doi: 10.1155/ijfo/1771084 (PMC11729517; doi:10.1155/ijfo/1771084)
Supplement: Supporting Information — Additional supporting information can be found online in the Supporting Information section.Figure S1 illustrates the nutritional analysis of the functionally enhanced sorghum bowl, as detailed in Table 2 and Section 3.2. The enhancement through roasting and the addition of flower powder significantly improves its nutritional value. Table S1 summarizes the phytochemical screening of the functionally enhanced sorghum bowl, discussed in Section 3.3 and Figure 3. The screening in aqueous, ethanol, chloroform, and acetone solvents reveals a strong presence of phytochemicals, indicating the enhanced bowl's robust phytochemical profile. Figure S2 depicts the antinutritional factors of the functionally enhanced sorghum bowl, elaborated in Table 3 and Section 3.5. Roasting significantly reduces antinutritional components such as total phenols, tannins, and phytic acid. Figure S3 presents a graph showing the toxicity analysis, total plate count, moisture content, and weight changes over 120 days, as part of the shelf life evaluation discussed in Sections 3.11 and 3.12 and detailed in Tables 5, 6, and 7. The toxicity analysis shows minimal toxicity, with fewer than three brine shrimp mortalities at the highest concentration (1500 μg/mL). The results for moisture content, weight changes, and total plate count confirm that the sorghum bowl maintains a shelf life exceeding 120 days. Figure S4 provides a graphical representation of the soil burial test for the functionally enhanced sorghum bowl, discussed in Section 3.13 and Figure 10. The standardized sorghum bowl fully degrades in wet topsoil within 12 days. [file 1771084.f1.docx]

**Optimizing Edible Sorghum Bowls: Effects of Roasting and Edible Flower Powder enhancement on Technological, Nutritional, Antioxidant, and Functional Properties**

Devatha Manivel^1^, Raajeswari Paramasivam^1^*, Swarup Roy^2^

**Supplementary File**

**Figure 1 – Nutritional Analysis of functionally enhanced sorghum bowl**

**Table – 1 Phytochemical Screening of functionally enhanced sorghum bowl**

| **Phytochemical Screening** | **SUB** | **SUHB** | **SURB** | **SRB** | **SRHB** | **SRRB** |
| --- | --- | --- | --- | --- | --- | --- |
| Alkaloids | - + + + | - + + + | + + + + | - - + + | - + + + | - + + + |
| Amino acids | + + + + | + + + + | + + + + | + + + + | + + + + | + + + + |
| Anthocyanins | - + + + | + + + + | - + + + | - - + + | - + + + | - - + + |
| Carbohydrate | + + + + | + + + + | + + + + | + + + + | + + + + | + + + + |
| Flavonoids | - + + + | + + + + | + + + + | - + + + | - + + + | - - + + |
| Glycosides | + + + + | - + - + | - + + + | - + + + | - + + + | - + + + |
| Phenol | + + + + | + + + + | + + + + | - + + + | + + + + | + + + + |
| Phytic acid | - + + + | + + + + | + + + + | - - + + | - - + + | - - - + |
| Saponin | - + + + | - + + + | + + + + | - + + + | - + + + | - + + + |
| Tannin | + + + + | ­+ + + + | + + + + | - - + + | - - + + | - - + + |
| Terpenoids | - + + + | + - + + | - + + + | - - + + | - - + + | - - + + |

Phytochemical screening of functionally enhanced sorghum bowl in the medium of aqueous, ethanol, chloroform and acetone

**Figure 2 Anti-nutritional factors of functionally enhanced sorghum bowl**

**Figure 3 a. Toxicity analysis, b. Total plate count over 120 days, c. Moisture Content over 120 days, d. Change in weight over 120 days of functionally enhanced sorghum bowl**

**Figure 4 Soil burial test of functionally enhanced sorghum bowl**
